# Web-based prediction models for predicting overall survival and cancer specific survival in lung metastasis of patients with thyroid cancer: a study based on the SEER database and a Chinese cohort

**DOI:** 10.7150/jca.103542

**Published:** 2024-11-04

**Authors:** Fangxu Yin, Song Wang, Ziying Jiang, Yunbin Tong, Lu Han, Wei Sun, Chengmeng Wang, Daqing Sun

**Affiliations:** 1 Department of Pediatric Surgery, Tianjin Medical University General Hospital, Tianjin, China.; 2 Department of Lung Cancer, Tianjin Medical University Cancer Institute and Hospital, National Clinical Research Center for Cancer, Key Laboratory of Cancer Prevention and Therapy, Tianjin's Clinical Research Center for Cancer, Tianjin Lung Cancer Center, Tianjin, China.

**Keywords:** Lung metastasis of thyroid cancer, Overall survival, Cancer-special survival, SEER database, Nomogram, Prognosis.

## Abstract

**Background:** The current high incidence of thyroid cancer (TC) is usually accompanied by poor prognosis of patients who also develop lung metastasis. Therefore, the present study aimed to develop a survival prediction model to guide clinical decision-making.

**Methods:** This study retrospectively analyzed 679 patients with TCLM from 2010 to 2015 using the Surveillance, Epidemiology, and End Results (SEER) database. The external validation cohort consisted of 48 patients from Tianjin Medical University General Hospital (TMUGP) and Tianjin Cancer Hospital (TCH). Cox proportional risk regression models were used to analyze prognostic influences on patients and the screened variables were used to build the survival prediction models. The present study used the C-index, time-dependent ROC curves, calibration curves, and decision curve analysis (DCA) were used to assess the performance of the nomogram models.

**Results:** The Cox proportional risk regression model analysis identified independent prognostic factors in patients with TCLM. In the training cohort, the C-index of the nomogram in predicting the overall survival (OS) was 0.813, cancer specific survival (CSS) was 0.822. The area under the receiver operator characteristics curve (AUC) values of the nomogram in prediction of the 1, 3, and 5-year OS were 0.884, 0.879 and 0.883. The AUC values for prediction of the 1, 3, and 5-year CSS were 0.887, 0.885 and 0.886. The C-index, time-dependent ROC curve, calibration curve, and DCA for the training group, internal validation group, and external validation group showed that the Nomogram had a clear advantage.

**Conclusion:** In this study, two new nomograms were constructed to predict the risk of TCLM patients. The nomograms can be applied in clinical practice to help clinicians assess patient prognosis.

## Introduction

TC is most prevalent tumor in the class of endocrine, head and neck tumors. It also accounts for about 1% of all systemic malignancies 1. Based on the SEER database, the incidence of TC reached 15.2 cases per 100,000 people in 2013, then slightly decrease to 13.7 cases per 100,000 people in 2017 2. Clinically, TC has an insidious onset, low malignancy, slow growth, and difficult early diagnosis, and a generally good prognosis with a 10-year survival rate usually exceeding 80% 3,4. The survival rate decreases in case metastases occur to distant organs or tissues such as lung, bone, and brain. Among these metastatic sites, lung metastases have an overall survival rate ranging from 25% to 75%, which is much lower compared to that of other metastatic sites 5.

During the 1994-2013 period, the number of patients with severe distant metastatic papillary thyroid cancer (PTC) increased by 2.9% per year 6. Of note, medullary thyroid carcinoma (MTC) is more prone to lymph node metastasis and distant metastasis than differentiated TC 7,8. The most common site of distant metastasis of anaplastic thyroid carcinoma (ATC) is the lung, followed by bone and brain, which account for less than 1% of cases, but the prognosis is poor. The 5-year survival rate was less than 10% 9. In terms of treatment, surgical resection is the main treatment modality for PTC and follicular thyroid carcinoma (FTC), followed by radioiodine ablation (RAI ablation) and thyroid hormone suppression therapy 10,11. Systemic radiotherapy and chemotherapy are rarely used, except in advanced cases that have failed to respond to conventional treatment. For TC patients with distant metastases, the commonly used drugs are kinase inhibitors 12. In addition to surgery, systemic chemotherapy with kinase inhibitors has been shown to be beneficial for refractory MTC 13. Tyrosine kinase inhibitors (TKIs) are the most commonly used treatments cabozantinib and vandetanib applied as first-line therapeutic agents. There is evidence that surgical resection after the introduction of TKI therapy prolongs the survival of patients, resulting in a 1-year overall survival of 94% 14.

Previous studies have shown that patient factors (age at diagnosis, gender, and treatment plan) and intrinsic tumor characteristics (histopathologic grade, local stage, lymph node status, and tumor size) are influential prognostic factors of patients with TCLM 15. However, the predictive value of these single factors is often limited. Currently, use of prognostic Nomogram to predict the OS and CSS of patients with tumors is more advantageous than the traditional TNM staging of the AJCC 16. However, only studies have developed predictive models to predict OS in IV stage of TCLM patients, lacking the prediction of CSS in TCLM patients and, most importantly, external validation. The present study was aimed to develop and validate a Nomogram prediction model for assessing patients with TCLM based on the SEER database and using multiple independent prognostic factors.

## Materials and Methods

### Ethics approval and consent to participate

The patients in the China and SEER database could not be identified, so the analyses and reporting of the data in our study were exempt from review by the Ethics Board of TMUGP and TCH, and the requirement for informed consent was waived because patient information was anonymized at every step of the study, including during data collation and statistical analysis.

### Data sources and patient selection

The SEER database is a large clinical database funded by the National Cancer Institute (NCI) which collects and publishes cancer incidence and survival data from 17 cancer registries.

We collected data on patients with TCLM registered in the SEER database from 2010 to 2015. Inclusion criteria were as follows: (i) the age of diagnosis was between 2010 and 2015; (ii) histological subtypes were classified into four categories, coded as follows:8340, 8341, 8342, 8344, 8260-PTC; 8330, 8331, 8335-FTC of follicular thyroid cancer; 8020, 8021 8030, 8032-ATC; 8510-MTC, all cases were staged according to the AJCC TNM classification (iii) no other confirmed tumors except for TCLM patients (iv) complete clinical and pathological data (v) complete follow-up information. Exclusion criteria: (i) patient age < 18 years at diagnosis; (ii) unknown race, marital status, surgery, bone metastasis, brain metastasis, liver metastasis; (iii) the follow-up time was 0 or unknown; (iv) patients with T0, Tx, N0 and NA stages screened according to the AJCC staging TNM classification. A total of 679 patients with TCLM were included through inclusion and exclusion criteria, of which 462 were randomized into the training group and the remaining 217 were randomized into the validation group. The OS was defined as the time interval from the date of diagnosis to the date of death or the last follow-up due to any cause, and CSS was defined as the time interval from the date of diagnosis to the date of death or the last follow-up of TCLM.

Clinical data of 48 patients with TCLM from 2010 to 2015 were retrospectively analyzed at TMUGP and TCH. We obtained clinicopathological parameters such as age, race, sex, marital status, T stage, N stage, surgery, radiotherapy, chemotherapy, bone metastasis, brain metastasis, liver metastasis, histologic type. Inclusion criteria were as follows:(i) the age of diagnosis was between 2010 and 2015; (ii) Histologic subtypes were classified as PTC, FTC, ATC and MTC; (iii) no other confirmed tumors except for TCLM patients (iv) complete clinical and pathological data; (v) complete follow-up information. Exclusion criteria: (i) patient age < 18 years at diagnosis; (ii) unknown race, marital status, surgery, bone metastasis, brain metastasis, liver metastasis and (iii) unknown survival records; (iv) patients with T0, Tx, N0 and NA stages screened according to the AJCC staging TNM classification. A follow-up was conducted through direct contact with patients or by telephone conversation with patients. In this study, the diagnosis of TCLM was used as the starting point for follow-up, with OS as the primary endpoint and CSS as the secondary endpoint. The follow-up ended on December 31, 2022.

### Construction and verification of Nomogram

The study was designed based on transparent reporting of a multivariable prediction model for individual prognosis or diagnosis (TRIPOD) 17. The following data were extracted through the SEER database: age, race, marital status, sex, bone metastasis, brain metastasis, liver metastasis, histological type, T-stage, N-stage, surgery, radiotherapy, and chemotherapy. The Cox univariate regression analysis was performed to screen out the variables with significant differences and included in the multifactorial regression. Independent prognostic factors were then integrated in the nomogram after identification. The scores of each independent prognostic factor were summed through transformation to assess the OS and CSS of patients with TCLM at 1, 3, and 5 years. Finally, the predictive power of the model was evaluated using the C-index and ROC curves, after which analysis of the calibration curve was used to assess the accuracy of the nomogram. Moreover, DCA was used to evaluate the potential benefits of nomograms.

### Risk stratification based on Nomogram

In this study, the risk scores of the 679 patients with TCLM were analyzed using X-tile software to obtain the best cut-off value and the patients were grouped by the cut-off value into high-risk, moderate-risk and low-risk groups. Lastly, the Kaplan-Meier survival was then used to assess the OS and CSS of TCLM patients.

### Statistical analysis

Statistical analysis of the data obtained in the present study was carried out using SPSS 22.0 and R language version 4.3.1. Count data were expressed as number of cases and rate (%) whereas the χ2 test was used for comparison between groups. The R statistical packages "scorecard", "rms", "survival" and "timeROC" were used to randomize groups, construct calibration plots and ROC curves, and build nomograms; "ggDCA" was used to plot DCA curves. shiny" and "DynNom" packages were used to develop a web-based survival calculator (https://www.shinyapps.io/) for predicting patient survival. Kaplan-Meier survival curves were used to describe the differences and associations between the two strata. All statistical tests were two-sided, and P-values <0.05 were considered statistically significant.

## Results

### Data sources and patient selection

**Figure 1:**
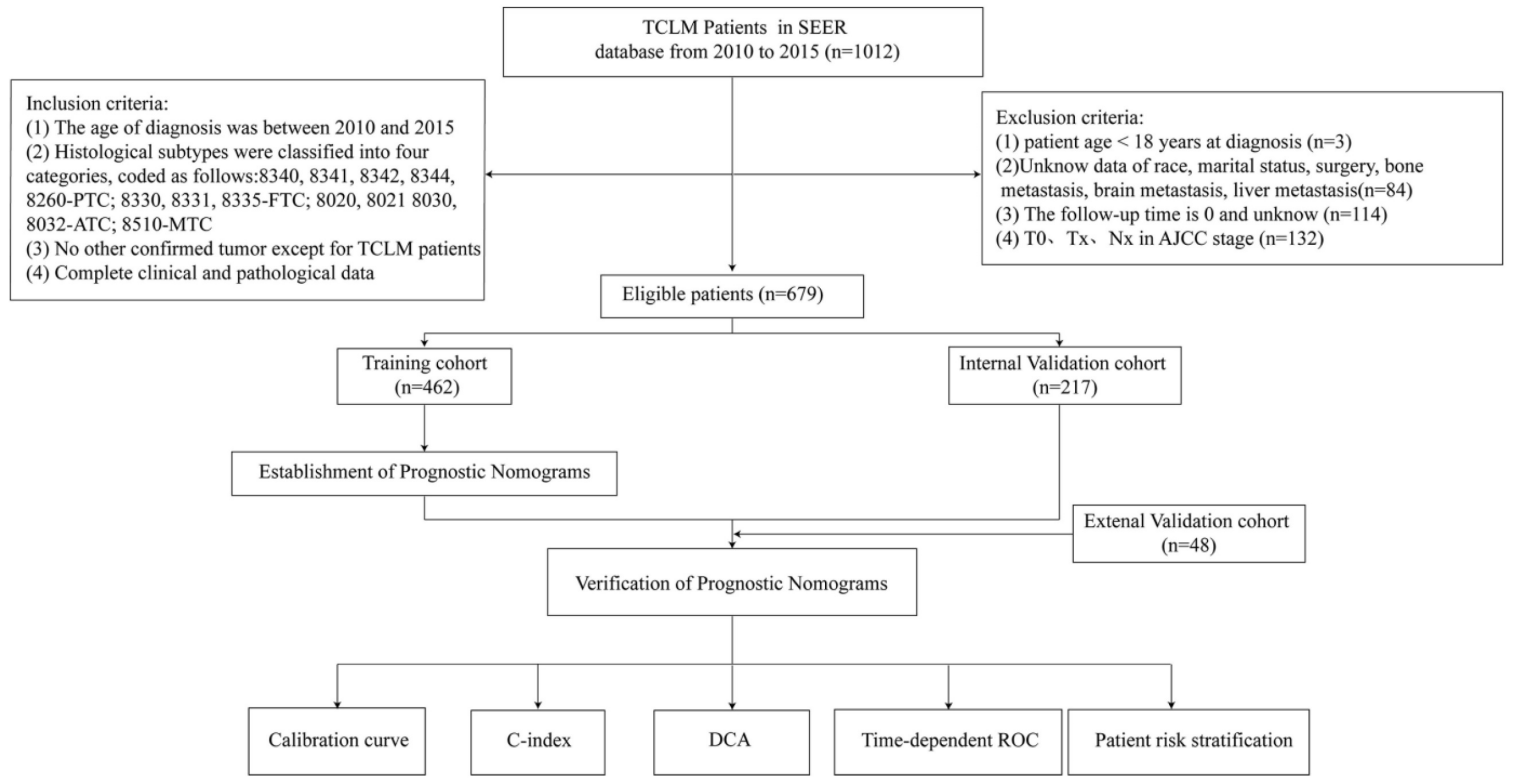
Flowchart of participant inclusion and exclusion.

Figure 1 is a flow chart of our research. The clinicopathological characteristics of the patients in the training and validation groups were as shown in Table 1. More than half of the patients were older than 60 years (65.8%). Further, it was found that 78.7% of the patients were white whereas 21.3% of them were black or other races. In addition, 51.9% and 48.1% of patients were female and male, respectively. Because of the already distant metastasis, results of TNM staging in the presents study showed a relatively high number of patients in stage T4 (60.4%) and stage N1 (68.0%). It was found that 21.9% of the patients were accompanied by bone metastasis whereas 5.6 and 5.6% of the patients had brain metastasis and liver metastasis, respectively. However, histological typing showed that 53.4%, 9.1%, 21.2% and 2.4% of patients had PTC, FTC, ATC, and MTC, respectively. In addition, it was found that 29.9%, 33.5%, and 76.6% of the patients did not receive surgery, radiation, and chemotherapy, respectively. In the external validation cohort, 77.1% of the patients were over 60 years of age, and all of them were from China. Females and males were 45.8% and 54.2%, respectively. Married patients were the most prevalent with 62.5%. The results of TNM staging showed a relatively high number of patients in stage T4 (52.3%) and N1 (62.5%). Bone metastasis was present in 27.1% of the patients, and brain metastasis and liver metastasis were present in 2.1% and 8.3% of the patients, respectively. Histological typing showed that 41.6%, 20.8%, 14.6%, and 4.2% of patients had PTC, FTC, ATC, and MTC, respectively. In addition, 39.6%, 29.2%, and 72.9% of patients did not undergo surgery, radiation, and chemotherapy, respectively.

### Establishment and validation of a prognostic nomogram

**Figure 2:**
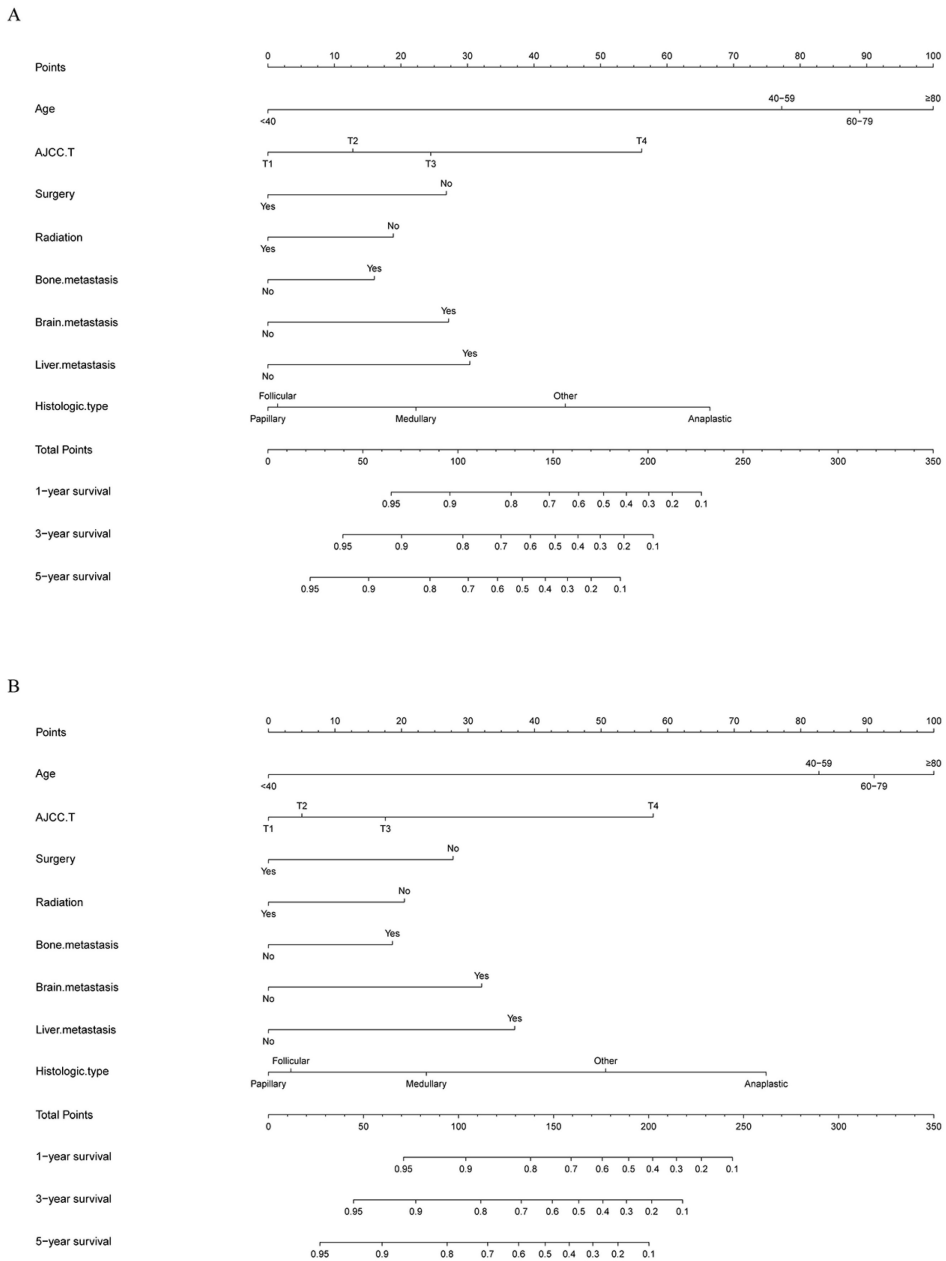
Nomograms for predicting 1-, 3-, and 5-year **(A)** OS and **(B)** CSS of patients with TCLM.

**Figure 3:**
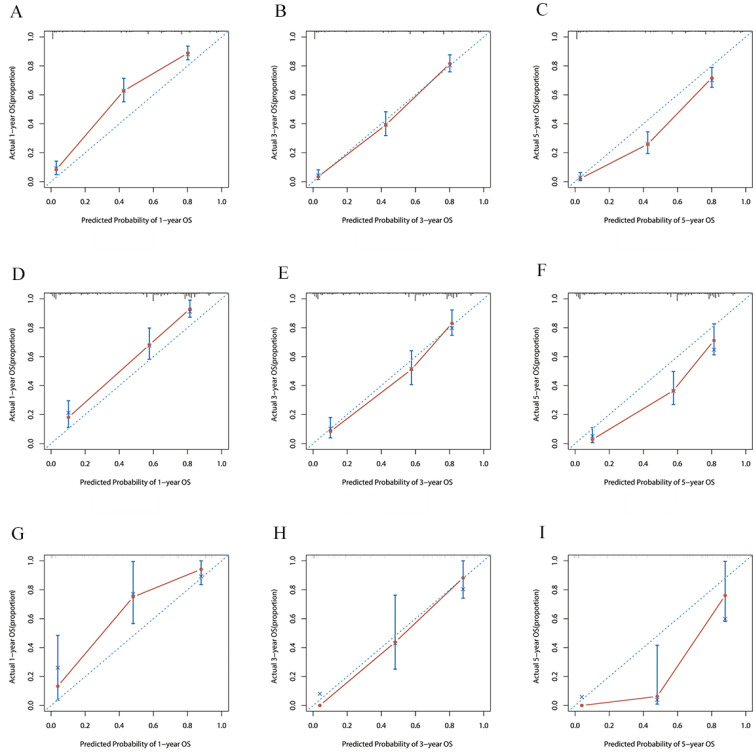
The calibration curves for predicting OS at **(A)** 1-year and **(B)** 3-year and **(C)** 5-year in the training group. The calibration curves for predicting OS at **(D)** 1-year **(E)** 3-year and **(F)** 5-year in the internal validation group. The calibration curves for predicting OS at **(G)** 1-year **(H)** 3-year and **(I)** 5-year in the external validation group.

**Figure 4:**
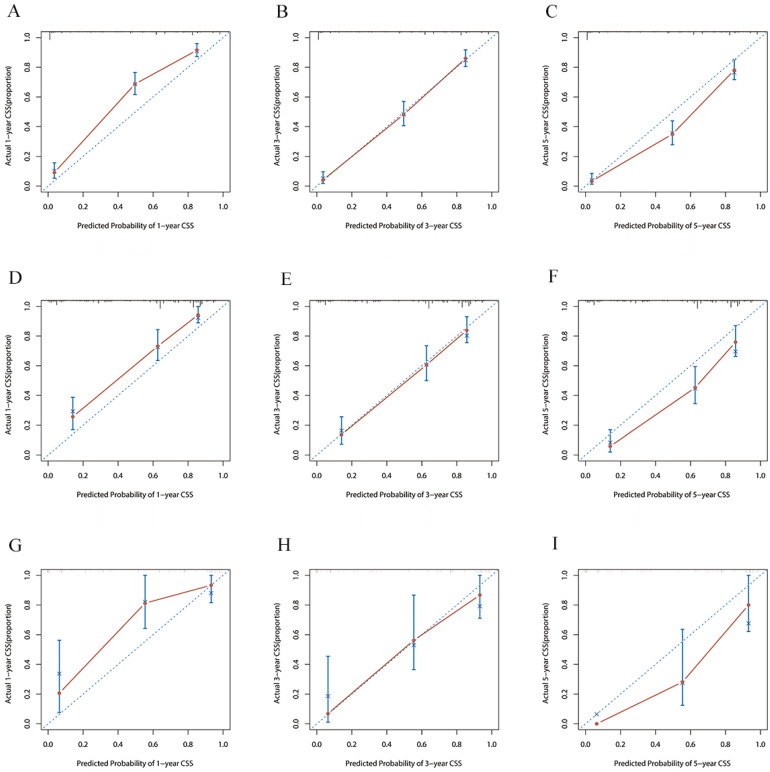
The calibration curves for predicting CSS at **(A)** 1-year and **(B)** 3-year and **(C)** 5-year in the training group. The calibration curves for predicting CSS at **(D)** 1-year **(E)** 3-year and **(F)** 5-year in the internal validation group. The calibration curves for predicting CSS at **(G)** 1-year **(H)** 3-year and **(I)** 5-year in the external validation group.

**Figure 5:**
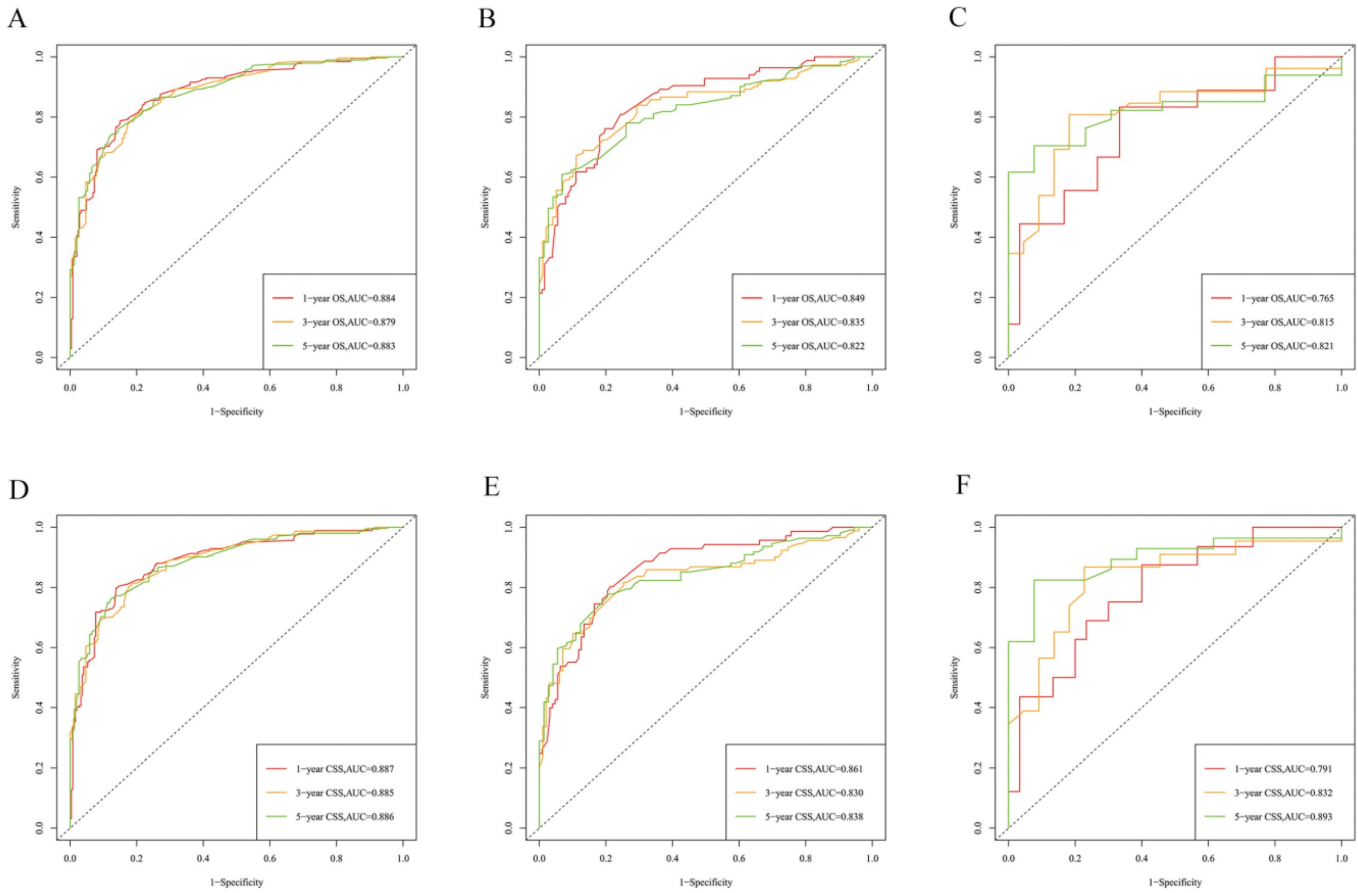
The time-dependent ROC curves of the nomogram predicting OS and CSS at **(A)** 1-year and 3-year and 5-year of OS in the training group, and at **(B)** 1-year 3-year and 5-year of OS in the internal validation group, **(C)** 1-year and 3-year and 5-year of CSS in the extenal validation group, and at **(D)** 1-year and 3-year and 5-year of CSS in the training group, and at **(E)** 1-year 3-year and 5-year of CSS in the internal validation group, **(F)** 1-year and 3-year and 5-year of CSS in the extenal validation group.

**Figure 6:**
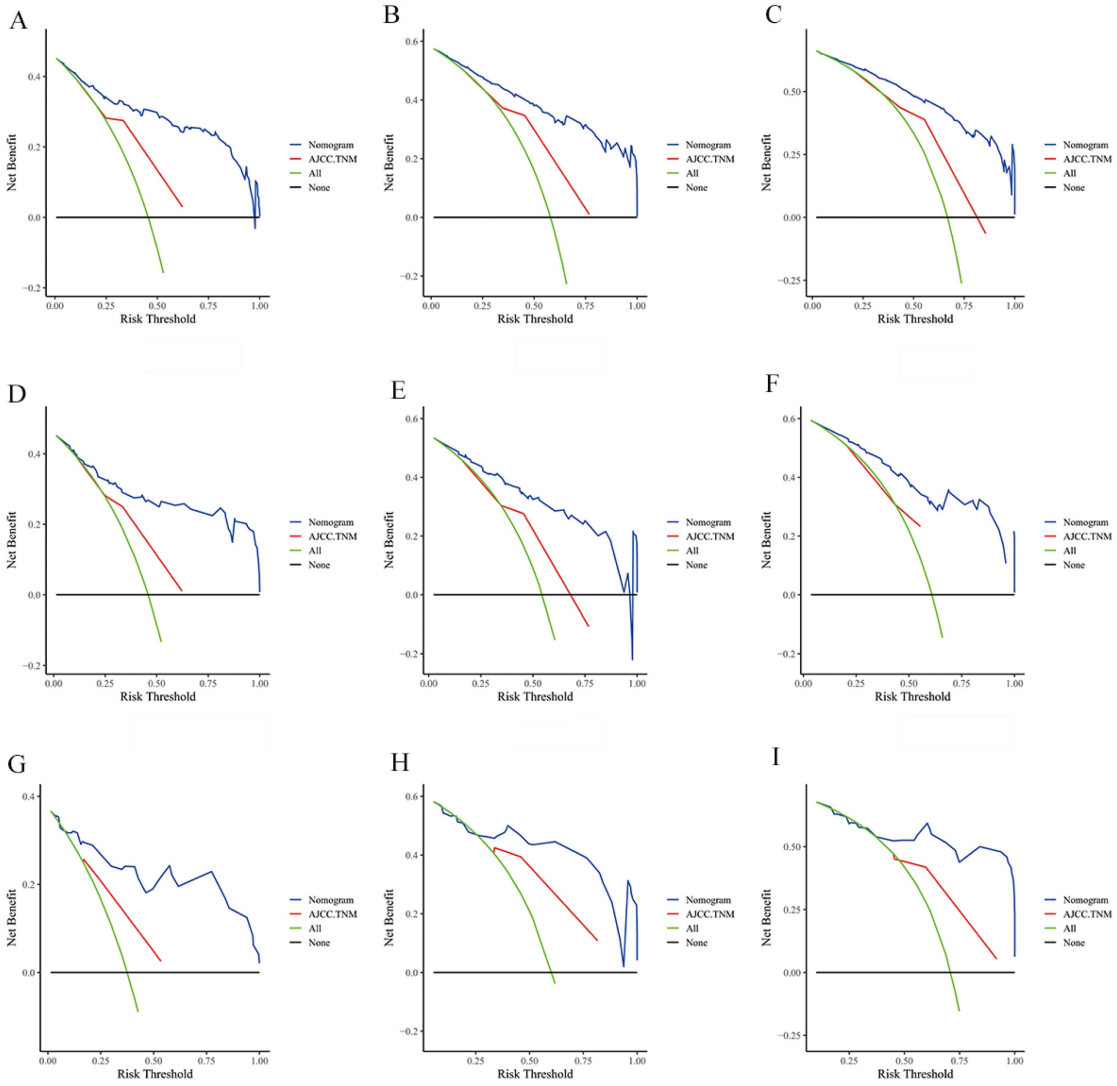
The DCA of the nomogram and AJCC.TNM for OS at **(A)** 1-year, **(B)** 3-year and **(C)** 5-year in the training group. The DCA of the nomogram and AJCC.TNM for OS at **(D)** 1-year, **(E)** 3-year and **(F)** 5-year in the internal validation group. The DCA of the nomogram and AJCC.TNM for OS at **(G)** 1-year, **(H)** 3-year and **(I)** 5-year in the extenal validation group.

**Figure 7:**
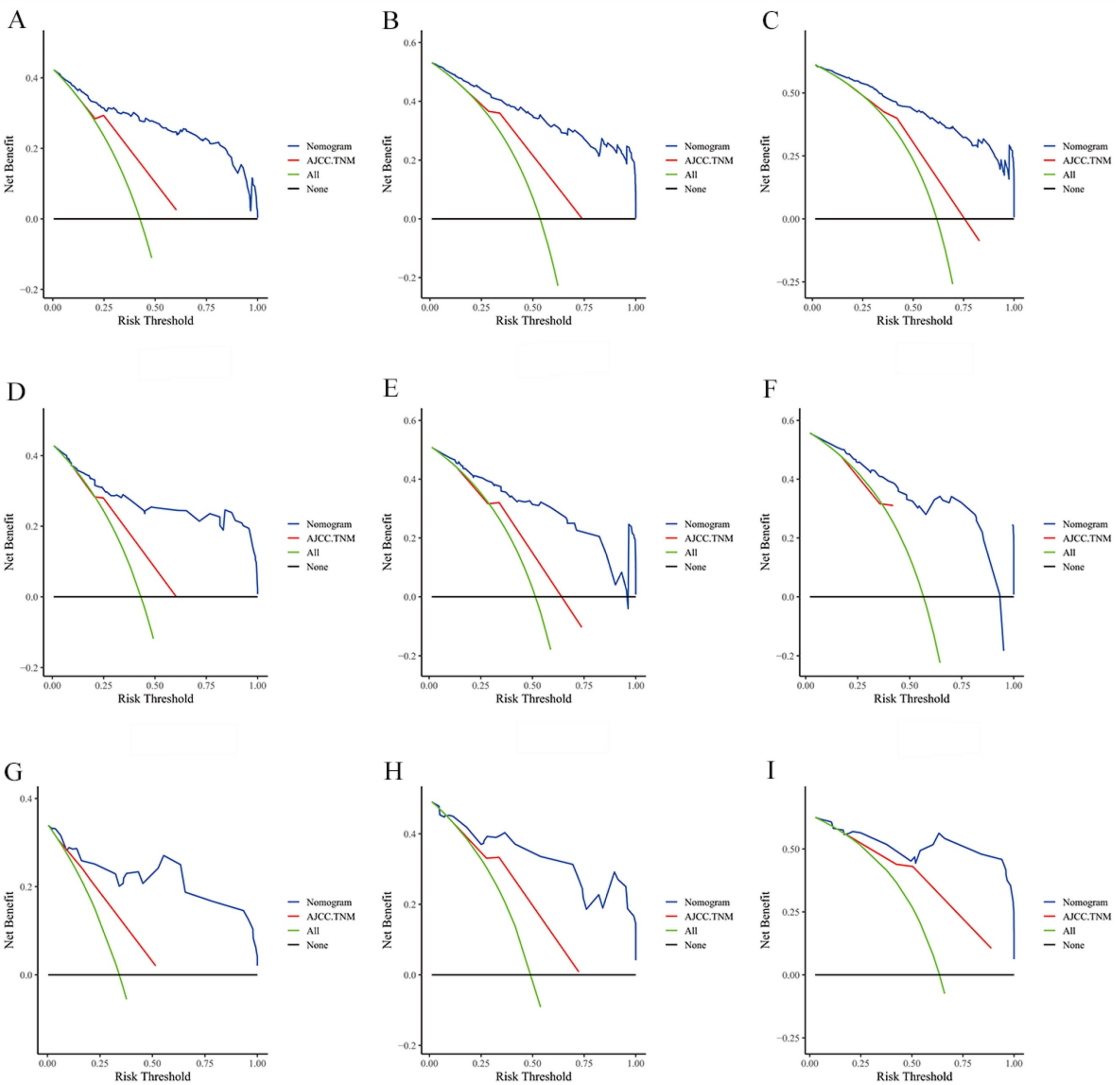
The DCA of the nomogram and AJCC.TNM for CSS at **(A)** 1-year, **(B)** 3-year and **(C)** 5-year in the training group. The DCA of the nomogram and AJCC.TNM for CSS at **(D)** 1-year, **(E)** 3-year and **(F)** 5-year in the internal validation group. The DCA of the nomogram and AJCC.TNM for CSS at **(G)** 1-year, **(H)** 3-year and **(I)** 5-year in the extenal validation group.

The risk ratios and univariate and multivariate Cox risk models are shown in Tables 2, 3. Further, eight independent prognostic factors for OS were screened including age, T stage, bone metastasis, brain metastasis, liver metastasis, histological type, surgery, and radiation. Eight independent prognostic factors for CSS were screened including age, T stage, bone metastasis, brain metastasis, liver metastasis, histological type, surgery, and radiation. These independent posterior factors were integrated for Nomogram construction and plotting, resulting in 1-, 3-, and 5-year OS and CSS prediction plots (Figure 2). After each influencing factor was individually compared to the score scale to derive a score, the resultant scores were summed to derive a total score and was then compared with the 1, 3, and 5-year overall survival rates corresponding to the total score scale at the bottom of the Nomogram. The validation results showed a C-index [OS: 0.813 (95% CI, 0.791-0.835) in the training group, 0.777 (95% CI, 0.744-0.810) in the internal validation group, and 0.827 (95% CI, 0.764-0.890) in the external validation group; CSS: 0.822 (95% CI, 0.799-0.845) in the training group, 0.786 (95% CI, 0.751-0.821) in the internal validation group, and 0.854 (95% CI, 0785-0.923) in the external validation group]. The calibration curves in the current study showed significant agreement between the survival probabilities predicted by the Nomogram and the actual observations in both the training and validation cohorts (Figure 3, Figure 4). Further, the time-dependent ROC curves were also used to assess the discriminatory ability of the Nomogram. In addition, values of the AUC for 1, 3, and 5 years were as presented in Figure 5 regarding OS [training group: 1-year OS 0.884 (95% CI, 0.852-0.915); 3-year OS 0.879 (95% CI, 0.849-0.910); 5-year OS 0.883 (95% CI, 0.852-0.914); internal validation group: 1-year OS 0.849 (95% CI, 0.800-0.902); 3-year OS 0.835 (95% CI, 0.781-0.890); 5-year OS 0.822 (95% CI, 0.767-0.878); external validation group: 1-year OS 0.765 (95% CI, 0.623-0.907); 3-year OS 0.815 (95% CI, 0.690-0.940); 5-year OS 0.821 (95% CI, 0.703-0.938)] and CSS [training group: 1-year CSS 0.887 (95% CI, 0.855-0.919); 3-year CSS 0.885 (95% CI, 0.855-0.916); 5-year CSS 0.886 (95% CI, 0.855-0.918); internal validation group: 1-year CSS 0.861 (95% CI, 0.808-0.915); 3-year CSS 0.830 (95% CI, 0.770-0.891); 5-year CSS 0.838 (95% CI, 0.781-0.894); external validation group: 1-year OS 0.791 (95% CI, 0.657-0.926); 3-year OS 0.832 (95% CI, 0.706-0.957); 5-year OS 0.893 (95% CI, 0.795-0.991)]. DCA decision curves can identify predictive models and help clinicians make better decisions. The superior net benefit suggests that the nomogram show more accurate values (Figure 6, Figure 7).

### Risk stratification

**Figure 8:**
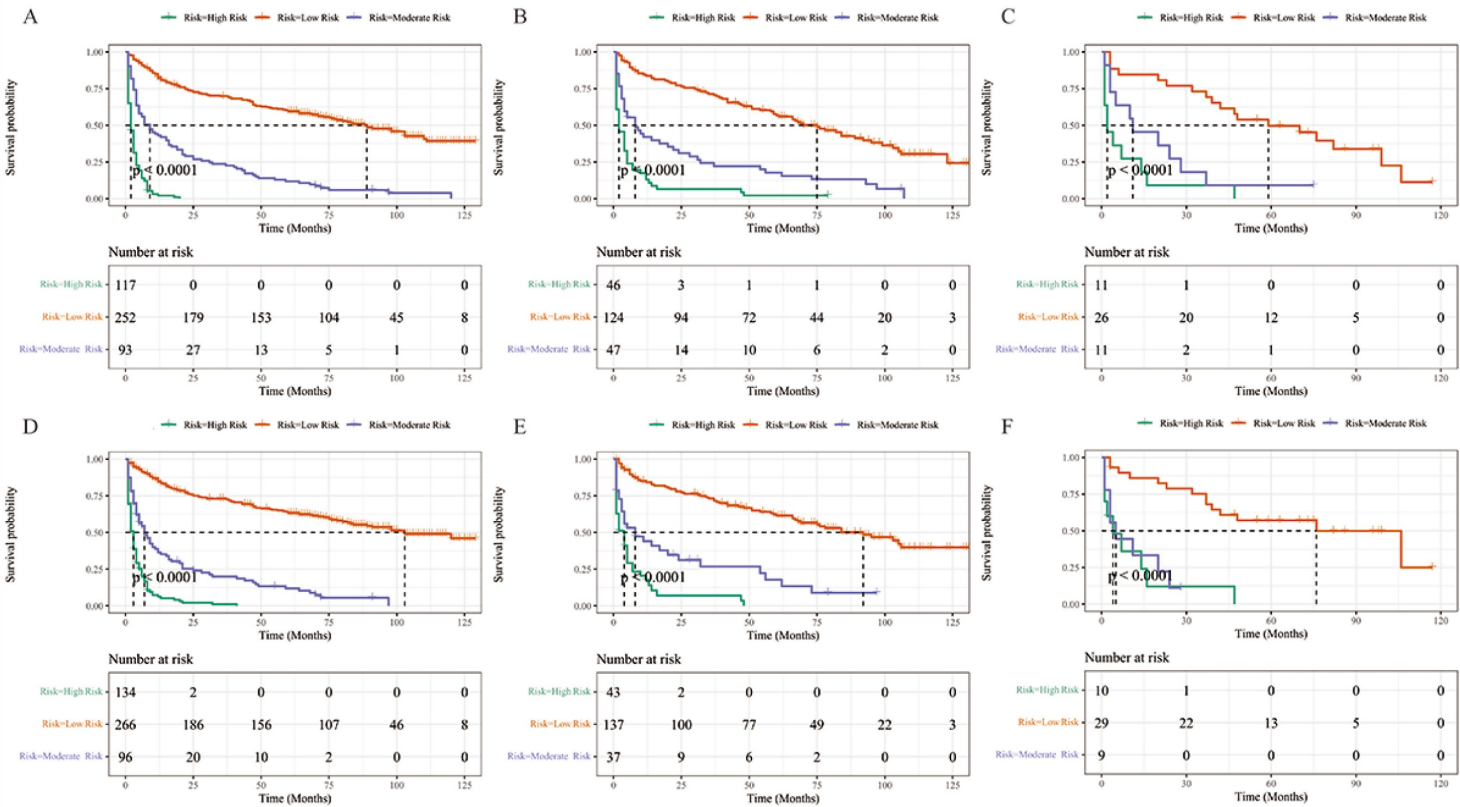
Performance of the nomograms in stratifying on the basis of risk points. **(A)** OS in the subgroups according to the risk stratification in the training cohort. **(B)** OS in the subgroups according to the risk stratification in the internal validation cohort. **(C)** OS in the subgroups according to the risk stratification in the extenal validation group. **(D)** CSS in the subgroups according to the risk stratification in the training cohort. **(E)** CSS in the subgroups according to the risk stratification in internal validation cohort. **(F)** CSS in the subgroups according to the risk stratification in the extenal validation cohort.

In this study, the risk scores for each variable were summed to obtain a total score for each TCLM patient, after which cutoff values were calculated using X-tile software; the cutoff values were 164 and 218 for OS and 178 and 244 for CSS. Therefore, TCLM patients with OS were categorized into low-risk (0-163), intermediate-risk (164-217) and high-risk (218-314) groups. In addition, CSS of TCLM patients was categorized into a low-risk group (0-177), an intermediate-risk group (178-243) and a high-risk group (244-337). Figure 8 shows the risk stratification of OS and CSS, and the survival analysis showed significant differences between these groups (OS in the training group, p < 0.0001; OS in the internal validation group, p < 0.0001; OS in the external validation group, p < 0.0001; CSS in the training group, p < 0.0001; CSS in the internal validation group, p < 0.0001; CSS in the external validation group, p < 0.0001). In the training group, 1-, 3-, and 5-year OS rates were respectively 82.5%, 69.8%, and 59.5% in the low-risk group, 43.0%, 23.7%, and 11.8% in the moderate-risk group, and 21.0%, 0%, and 0% in the high-risk group. The 1-, 3-, and 5-year CSS rates were respectively 84.7%, 73.1%, and 63.3% in the low-risk group, 36.4%, 20.0%, and 11.9% in the moderate-risk group, and 6.3%, 1.0%, and 0% in the high-risk group. In the internal validation group, 1-, 3-, and 5-year OS rates were respectively 83.8%, 71.4%, and 56.8% in the low-risk group, 42.0%, 24.3%, and 17.7% in the moderate-risk group, and 13.0%, 6.5%, and 2.2% in the high-risk group. The 1-, 3-, and 5-year CSS rates were respectively 84.2%, 73.2%, and 61.5% in the low-risk group, 44.1%, 26.7%, and 17.8% in the moderate-risk group, and 17.5%, 7.0%, and 0% in the high-risk group. In the external validation group, 1-, 3-, and 5-year OS rates were respectively 84.6%, 73.1%, and 49.7% in the low-risk group, 45.5%, 18.2%, and 9.1% in the moderate-risk group, and 27.3%, 9.1%, and 0% in the high-risk group. The 1-, 3-, and 5-year CSS rates were respectively 85.9%, 75.2%, and 57.1% in the low-risk group, 33.3%, 0%, and 0% in the moderate-risk group, and 36.0%, 12.0%, and 0% in the high-risk group. In the current study, the Nomogram were effectively implemented to stratify patients with TCLM.

### Dynamic web-based survival rate calculator

Based on the nomograms we created, we developed two web servers to predict the OS (https://houchong.shinyapps.io/tclmos/) and CSS (https://houchong.shinyapps.io/tclmcss/) of TCLM patients. By inputting information about TCLM patients, the intertemporal survival probability of patients can be easily predicted, which can better assist clinical efforts.

## Discussion

Some clinical features such as age, gender, postoperative thyroglobulin (Tg) level, and tumor size have been reported to be the risk factors causing pulmonary metastasis in patients with TC 18. Lung is the most common site of metastasis in differentiated TC. It is followed by bone and rarely brain and liver 19. Therefore, accurate prediction of the survival rate of patients with TCLM disease is of utmost importance for effective clinical management and medical decision making. Nomogram is a common tool for medical prognosis. In the present study, a more complete evaluation system was hence constructed. It assisted clinicians in decision making during patient treatment.

Interestingly, although patient race and gender were found to be important factors influencing the prognosis of TC 20, race and gender were not identified as independent prognostic factors in our univariate analysis. With an increased proportion of risk occurring with increasing age, age is an important factor in prognosis 21. A previous study conducted by Li *et al.* evaluated radioiodine for distant metastases in TC and it was concluded that old patients have a poorer prognosis than young patients with TC 13. Similarly, the present study found that older patients with TCLM had a poor prognosis, and the risk ratios increased with age (<40, HR=1; 40-59, HR=5.882; 60-79, HR= 7.670; ≧80, HR=9.858), and patients aged ≧80 years were found to have a lower OS and CSS. This may be due to the fact that elderly people tend to have poor compliance as compared with younger people. In addition, the inability of elderly people to receive timely treatment or inappropriate treatment can also lead to their poor prognosis.

There is more agreement on the effect of histologic grade, T-stage, and N-stage on the survival prognosis of patients with TCLM. In clinical setting, extent of tumor invasion is commonly used to predict the prognosis of patients with tumors 22. In a study Tong *et al.* 23 assessed independent risk factors of overall survival and cancer-specific survival of patients with TC and bone metastases. It was reported that patients with tumor diameter ≤8 cm had a good prognosis whereas those with tumor diameter >8 cm had a poor prognosis. The present study confirmed that T-stage affects the survival prognosis of patients with TCLM, and the risk ratio was higher for T4 stage (HR=3.665, 95% CI 1.904-7.057, P<0.001). It is easy to cause clinical misdiagnosis and underdiagnosis because there are no specific clinical symptoms in the early stage of TC. Further, the malignant tumor is often larger when patients are found, the risk of invasion of surrounding tissues, occult foci, multicentric cancer foci, envelope infiltration, and lymph node metastasis are greatly increased at that time. This makes surgery more difficult and may cause residual lesions because of the difficulty in achieving complete removal during surgery, thus leading to recurrence of the cancer 24. Previous studies have concluded that N stage is one of the factors that have a great impact on prognosis of patients 23,25, and N stage should have been included as an independent prognostic risk factor for patients with TCLM. In our study, there was no correlation between N-stage and TCLM patients, which is similar to the findings of Wang *et al.* 26. The reason for not including it in the present study is because the sample size was not sufficient. Furthermore, the patients with TC have a better prognosis, whereas patients with TCLM are very rare.

Thyroid cancer is currently treated mainly by surgery, including the clearance of the primary site of cancer and the involved tissues as well as the metastatic lymph nodes 27. In the current study, surgery was associated with good prognosis in patients with TC. Furthermore, retrospective studies and randomized trials have shown that primary tumor surgery may improve cancer survival by reducing the tumor load of patients, RAI therapy is the ideal treatment for patients with TC 10. In addition, it was noted that all other types of TC are less sensitive to radiotherapy with an exception of undifferentiated TC and thus the external radiotherapy is the main treatment for undifferentiated cancers, external radiotherapy can improve the 10-year disease-free survival rate 28. Our results suggest that radiotherapy influences the survival of patients, but chemotherapy is not an independent factor influencing survival in TCLM patients. This is probably because the SEER database does not collect information on adjuvant chemotherapy and molecularly targeted therapies for patients.

In usual clinical practice, the more sites of cancer metastases, the prognosis for patients was worse 29. Zhong *et al.* 30 studied a study of TCLM versus bone, brain, or liver metastases found that there was a significant difference in prognosis between lung metastases alone compared with multiorgan metastases including lung metastasis. And that the concurrent occurrence of two metastatic lesions with a better prognosis did not improve the prognosis of the patients but rather was worse than that of patients with metastasis alone. In a study on bone metastasis of thyroid cancer, liver metastasis was reported to be an independent risk factor 23. Liver metastasis and bone metastasis were found to be independent prognostic factors for lung metastasis in stage IV thyroid cancer in a study predicting lung metastasis in Stage IV Thyroid Cancer 26. Our study found that bone metastasis, brain metastasis, and liver metastasis were all independent prognostic factors in patients with TCLM, and it was hypothesized that organ failure due to organ involvement of these metastases may be a possible cause of death in some patients with TCLM 31,32.

It was evident that different pathological types of TC have different biological manifestations, whereby ATC have the worst prognosis, PTC have the best prognosis, followed by FTC and MTC, which also validates the rapid progression of ATC with rapid involvement of adjacent tissues and organs 28. Previous studies have also confirmed that ATC is one of the highly aggressive solid tumors, accounting for approximately 50% of deaths due to TC annually, with a median survival of 5 months and a 1-year survival rate of less than 20% 33,34. The present study showed that ATC were associated with poor prognosis, which is generally in agreement with the findings of previous studies 35.

Previously, Zhong *et al.* 30 and Liu *et al.* 36 only analyzed the significant influencing factors of TCLM patients by SEER database and did not develop a model to predict the prognosis of patients. Later, Wang *et al.* developed a survival prediction model for IV stage of TCLM patients. Tong *et al.* 23 included 242 TCBM patients from 2010 to 2016 in the SEER database for patients with thyroid cancer bone metastases (TCBM) and developed OS and CSS prediction models for TCBM, which can help to make accurate judgments in clinical practice. Shi *et al.* 37 used a random forest model to develop an accurate prognostic model for predicting OS and CSS at 3 and 5 years for TCBM. We're different from Shi. We utilized a nomogram to accurately predict OS and CSS in TCLM. Kuang *et al.* 38 analyzed risk factors for lung metastasis of differentiated thyroid cancer in children and developed a clinical predictive model. In contrast, we studied TCLM across the entire age range, with a wider range and applied an external validation cohort for secondary validation of the model, which was found to be highly accurate.

There are several notable advantages in this study. First, to our knowledge, this study is the first to develop and validate two nomograms for predicting OS and CSS in patients with TCLM. Our study, which is multicenter and includes the SEER database, TMUGP and TCH. It's the first to use externally validated data to test the accuracy of the model, and excitingly, after external validation, our model shows excellent efficacy. Second, this study incorporates risk factors other than TNM stage that affect the prognosis of patients with TCLM, and we have developed reliable nomograms for patients with TCLM while also classifying them into high, moderate and low risk groups. The definition of risk stratification can provide a basis for prognostic judgment and individualized treatment plans to some extent. Thirdly, the predictive models we developed obtained higher AUC values compared to clinical individual factors. In the DCA curve, our two nomograms exhibited a good degree of clinical benefit than TNM staging. Finally, we have created TCLM's OS and CSS web calculators, which predict the survival rate by selecting various variables from online websites and setting a time. The use of predictive calculators is extremely convenient, and increasing the number of predictive models does not create a burden, making the focus of building models on improving the accuracy of the model. With the update of the SEER database and the improvement of patient clinical information, 679 patients with TCLM from 2010 to 2015 were included in this study, and then OS and CSS prediction models applicable to patients with TCLM were constructed. Among the metastatic sites, the overall survival rate of pulmonary metastases was much lower than that of other metastatic sites, and the lung was the most common metastatic site in differentiated TC, and the accuracy of the prediction model we built was high.

However, the prediction model developed in the present also had some limitations. First, the Nomogram was constructed from retrospectively collected data. Therefore, this could lead to a potential risk of selection bias. Second, some other important prognostic variables such as RET mutation status and calcitonin levels were not available in the SEER database. Finally, the SEER database does not have specific information about treatment options and postoperative examinations among others hence the undescribed factors may also affect the overall results.

## Conclusion

In conclusion, our model showed that TCLM patients with aged ≥ 80 years, advanced stage, anaplastic cancer, no surgery and no radiation, bone metastasis, brain metastasis and liver metastasis had poor prognosis. In this study, we successfully constructed and validated visualized nomograms predicting 1-year, 3-year and 5-year OS and CSS in TCLM patients, which can be used as an auxiliary predictive tool in clinical practice and to develop accurate individualized plans for patients.

## Figures and Tables

**Table 1:** Characteristics of patients with TCLM in the training and validation group.

Characteristics	Training cohort	Internal validation cohort	Overall	External validation cohort	T vs IV	T vs EV
	(n = 462)	(n = 217)	(n = 679)	(n = 48)	P	P
	No. of patients (%)	No. of patients (%)	No. of patients (%)	No. of patients (%)		
Age					0.249	0.031
<40	53(11.5)	19(8.8)	72(10.6)	2(4.2)		
40-59	105(22.7)	40(18.4)	145(21.4)	9(18.7)		
60-79	245(53.0)	122(56.2)	367(54.0)	24(50.0)		
≧80	59(12.8)	36(16.6)	95(14.0)	13(27.1)		
Race					0.055	<0.010
White	364(78.7)	162(74.7)	526(77.5)	0(0.0)		
Black	28(6.1)	25(11.5)	53(7.8)	0(0.0)		
Other1	70(15.2)	30(13.8)	100(14.7)	48(100.0)		
Sex					0.887	0.420
Female	240(51.9)	114(52.5)	354(52.1)	22(45.8)		
male	222(48.1)	103(47.5)	325(47.9)	26(54.2)		
Marital status					0.809	0.410
Married	244(52.8)	119(54.8)	363(53.5)	30(62.5)		
Unmarried	108(23.4)	46(21.2)	154(22.6)	8(16.7)		
Other2	110(23.8)	52(24.0)	162(23.9)	10(20.8)		
AJCC T					0.103	0.226
T1	28(6.1)	14(6.5)	42(6.2)	3(6.3)		
T2	25(5.4)	23(10.5)	48(7.1)	8(16.6)		
T3	130(28.1)	57(26.3)	187(27.5)	10(20.8)		
T4	279(60.4)	123(56.7)	402(59.2)	27(56.3)		
AJCC N					0.951	0.442
N0	148(32.0)	69(31.8)	217(32.0)	18(37.5)		
N1	314(68.0)	148(68.2)	462(68.0)	30(62.5)		
Bone metastasis					0.455	0.409
Yes	101(21.9)	42(19.4)	143(21.1)	13(27.1)		
No	361(78.1)	175(80.6)	536(78.9)	35(72.9)		
Brain metastasis					0.101	0.297
Yes	26(5.6)	6(2.8)	32(4.7)	1(2.1)		
No	436(94.4)	211(97.2)	647(95.3)	47(97.9)		
Liver metastasis					0.188	0.448
Yes	26(5.6)	18(8.3)	44(6.5)	4(8.3)		
No	436(94.4)	199(91.7)	635(93.5)	44(91.7)		
Histologic Type					0.526	0.055
Papillary	247(53.4)	112(51.7)	359(52.9)	20(41.6)		
Follicular	42(9.1)	23(10.6)	65(9.6)	10(20.8)		
Anaplastic	98(21.2)	35(16.1)	133(19.6)	7(14.6)		
Medullary	11(2.4)	17(7.8)	28(4.1)	2(4.2)		
Other3	64(13.9)	30(13.8)	94(13.8)	9(18.8)		
Surgery					0.453	0.165
Yes	324(70.1)	146(67.3)	470(69.2)	29(60.4)		
No	138(29.9)	71(32.7)	209(30.8)	19(39.6)		
Radiation					0.465	0.539
Yes	307(66.5)	138(63.6)	445(65.5)	34(70.8)		
No	155(33.5)	79(36.4)	234(34.5)	14(29.2)		
Chemotherapy					0.490	0.566
Yes	108(23.4)	56(25.8)	164(24.2)	13(27.1)		
No	354(76.6)	161(74.2)	515(75.8)	35(72.9)		

HR, hazard ratio; 95 CI, 95% confidence interval; Other1, including Asian or Pacific Islander and American Indian/Alaska Native; Other2, including separated, divorced and widowed; Other3, including Pleomorphic carcinoma, Giant cell carcinoma, Spindle cell carcinoma, Pseudosarcomatous carcinoma, Non-small cell carcinoma, Squamous cell carcinoma, Neuroendocrine carcinoma, Oxyphilic adenocarcinoma, Clear cell adenocarcinoma, Insular carcinoma, Nonencapsulated sclerosing carcinoma, Spindle cell sarcoma, Hemangiosarcoma, Acinar cell carcinoma and Epithelioid leiomyosarcoma; T,Training cohort; IV, Internal validation cohort; EV, External validation cohort.

**Table 2:** Overall Survival Univariate analysis and Multivariate analysis of the training cohort

Characteristics	Overall Survival Univariate analysis			Overall Survival Multivariate analysis		
	HR	95%CI	P-value	HR	95%CI	P-value
Age						
<40	1	[Reference]		1	[Reference]	
40-59	7.418	3.562-15.448	<0.001	5.882	2.761-12.528	<0.001
60-79	9.594	4.715-19.521	<0.001	7.670	3.644-16.147	<0.001
≧80	18.471	8.739-39.043	<0.001	9.858	4.429-21.945	<0.001
Race						
White	1	[Reference]				
Black	0.831	0.509-1.358	0.460			
Other^1^	1.208	0.901-1.619	0.207			
Sex						
Female	1	[Reference]				
male	1.017	0.819-1.261	0.881			
Marital status						
Married	1	[Reference]				
Unmarried	0.543	0.400-0.737	<0.001			
Other^2^	1.255	0.979-1.608	0.073			
AJCC T						
T1	1	[Reference]		1	[Reference]	
T2	1.634	0.530-3.186	0.252	1.379	0.589-3.230	0.460
T3	1.672	0.573-2.115	0.129	1.781	0.910-3.483	0.092
T4	5.249	1.836-6.002	<0.001	3.665	1.904-7.057	<0.001
AJCC N						
N0	1	[Reference]				
N1	0.901	0.718-1.130	0.366			
Bone metastasis						
Yes	1	[Reference]		1	[Reference]	
No	0.540	0.422-0.691	<0.001	0.692	0.528-0.908	0.008
Brain metastasis						
Yes	1	[Reference]		1	[Reference]	
No	0.374	0.249-0.561	<0.001	0.544	0.356-0.830	0.005
Liver metastasis						
Yes	1	[Reference]		1	[Reference]	
No	0.360	0.240-0.541	<0.001	0.530	0.342-0.821	0.005
Histologic Type						
Papillary	1	[Reference]		1	[Reference]	
Follicular	1.379	0.935-2.035	0.105	1.051	0.689-1.602	0.818
Anaplastic	7.556	5.608-10.179	<0.001	4.204	2.975-5.941	<0.001
Medullary	1.709	0.838-3.487	0.141	1.655	0.780-3.511	0.189
Other^3^	2.512	1.830-3.449	<0.001	2.657	1.885-3.746	<0.001
Surgery						
Yes	1	[Reference]		1	[Reference]	
No	4.223	3.337-5.345	<0.001	1.806	1.380-2.364	<0.001
Radiation						
Yes	1	[Reference]		1	[Reference]	
No	2.076	1.662-2.592	<0.001	1.513	1.184-1.935	0.001
Chemotherapy						
Yes	1	[Reference]				
No	0.379	0.297-0.484	<0.001			

HR, hazard ratio; 95 CI, 95% confidence interval; Other1, including Asian or Pacific Islander and American Indian/Alaska Native; Other2, including separated, divorced and widowed; Other3, including Pleomorphic carcinoma, Giant cell carcinoma, Spindle cell carcinoma, Pseudosarcomatous carcinoma, Non-small cell carcinoma, Squamous cell carcinoma, Neuroendocrine carcinoma, Oxyphilic adenocarcinoma, Clear cell adenocarcinoma, Insular carcinoma, Nonencapsulated sclerosing carcinoma, Spindle cell sarcoma, Hemangiosarcoma, Acinar cell carcinoma and Epithelioid leiomyosarcoma; T,Training cohort; IV, Internal validation cohort; EV, External validation cohort.

**Table 3:** Cancer-specific Survival Univariate analysis and Multivariate analysis of the training cohort.

Characteristics	Cancer-specific Survival Univariate analysis			Cancer-specific Survival Multivariate analysis		
	HR	95%CI	P-value	HR	95%CI	P-value
Age						
<40	1	[Reference]		1	[Reference]	
40-59	7.722	3.535-16.869	<0.001	5.816	2.600-13.014	<0.001
60-79	9.030	4.226-19.298	<0.001	6.845	3.090-15.166	<0.001
≧80	17.102	7.689-38.042	<0.001	8.211	3.499-19.266	<0.001
Race						
White	1	[Reference]				
Black	0.763	0.445-1.309	0.326			
Other^1^	1.117	0.812-1.538	0.496			
Sex						
Female	1	[Reference]				
male	0.921	0.732-1.159	0.484			
Marital status						
Married	1	[Reference]				
Unmarried	0.543	0.390-0.755	<0.001			
Other^2^	1.333	1.027-1.731	0.031			
AJCC T						
T1	1	[Reference]		1	[Reference]	
T2	1.340	0.532-3.377	0.535	1.126	0.441-2.877	0.804
T3	1.412	0.696-2.862	0.339	1.474	0.720-3.016	0.288
T4	5.162	2.643-10.082	<0.001	3.434	1.718-6.864	<0.001
AJCC N						
N0	1	[Reference]				
N1	0.967	0.757-1.234	0.785			
Bone metastasis						
Yes	1	[Reference]		1	[Reference]	
No	0.514	0.397-0.665	<0.001	0.664	0.500-0.883	0.005
Brain metastasis						
Yes	1	[Reference]		1	[Reference]	
No	0.340	0.226-0.512	<0.001	0.504	0.329-0.774	0.002
Liver metastasis						
Yes	1	[Reference]		1	[Reference]	
No	0.328	0.218-0.494	<0.001	0.483	0.310-0.752	0.001
Histologic Type						
Papillary	1	[Reference]		1	[Reference]	
Follicular	1.446	0.952-2.198	0.084	1.100	0.699-1.731	0.681
Anaplastic	8.534	6.243-11.667	<0.001	4.600	3.206-6.600	<0.001
Medullary	1.807	0.842-3.878	0.129	1.661	0.739-3.732	0.219
Other^3^	2.669	1.900-3.750	<0.001	2.810	1.943-4.064	<0.001
Surgery						
Yes	1	[Reference]		1	[Reference]	
No	4.215	3.287-5.405	<0.001	1.743	1.315-2.311	<0.001
Radiation						
Yes	1	[Reference]		1	[Reference]	
No	2.057	1.624-2.605	<0.001	1.544	1.187-2.007	0.001
Chemotherapy						
Yes	1	[Reference]				
No	0.342	0.265-0.440	<0.001			

HR, hazard ratio; 95 CI, 95% confidence interval; Other^1^, including Asian or Pacific Islander and American Indian/Alaska Native; Other^2^, including separated, divorced and widowed; Other^3^, including Pleomorphic carcinoma, Giant cell carcinoma, Spindle cell carcinoma, Pseudosarcomatous carcinoma, Non-small cell carcinoma, Squamous cell carcinoma, Neuroendocrine carcinoma, Oxyphilic adenocarcinoma, Clear cell adenocarcinoma, Insular carcinoma, Nonencapsulated sclerosing carcinoma, Spindle cell sarcoma, Hemangiosarcoma, Acinar cell carcinoma and Epithelioid leiomyosarcoma; T,Training cohort; IV, Internal validation cohort; EV, External validation cohort.
